# Intuitive visualizations of pitch and loudness in speech

**DOI:** 10.3758/s13423-015-0934-0

**Published:** 2015-09-14

**Authors:** Rebecca S. Schaefer, Lilian J. Beijer, Wiel Seuskens, Toni C. M. Rietveld, Makiko Sadakata

**Affiliations:** Health, Medical and Neuropsychology Unit, Institute of Psychology, Leiden University, Leiden, The Netherlands; Leiden Institute for Brain and Cognition (LIBC), Leiden University, Leiden, The Netherlands; Sint Maartenskliniek Research, Nijmegen, The Netherlands; Donders Institute for Brain, Cognition and Behaviour, Centre for Cognition, Nijmegen, The Netherlands; Department of Linguistics, Radboud University Nijmegen, Nijmegen, The Netherlands; The Institute for Logic, Language and Computation, University of Amsterdam, Amsterdam, The Netherlands

**Keywords:** Audio-visual processing, Visualizing sound, Speech therapy, Feedback learning

## Abstract

Visualizing acoustic features of speech has proven helpful in speech therapy; however, it is as yet unclear how to create intuitive and fitting visualizations. To better understand the mappings from speech sound aspects to visual space, a large web-based experiment (*n* = 249) was performed to evaluate spatial parameters that may optimally represent pitch and loudness of speech. To this end, five novel animated visualizations were developed and presented in pairwise comparisons, together with a static visualization. Pitch and loudness of speech were each mapped onto either the vertical (*y*-axis) or the size (*z*-axis) dimension, or combined (with size indicating loudness and vertical position indicating pitch height) and visualized as an animation along the horizontal dimension (*x*-axis) over time. The results indicated that firstly, there is a general preference towards the use of the *y*-axis for both pitch and loudness, with pitch ranking higher than loudness in terms of fit. Secondly, the data suggest that representing both pitch and loudness combined in a single visualization is preferred over visualization in only one dimension. Finally, the *z*-axis, although not preferred, was evaluated as corresponding better to loudness than to pitch. This relation between sound and visual space has not been reported previously for speech sounds, and elaborates earlier findings on musical material. In addition to elucidating more general mappings between auditory and visual modalities, the findings provide us with a method of visualizing speech that may be helpful in clinical applications such as computerized speech therapy, or other feedback-based learning paradigms.

## Introduction

Visualizing sounds is useful in a range of learning situations in which a visual representation can give information about the sound produced, e.g., speech (Demenko, Wagner & Clywik, 2010; Watanabe, Tomishige & Nakatake, 2000) or music (Dixon, 2007; Hoppe, Sadakata, & Desain, 2006; McLeod, 2008; Sadakata, Hoppe, Brandmeyer, Timmers, & Desain, 2008; Stowell & Plumbley, 2007). It is also increasingly common for software dealing with audio signals to support acoustic analyses by providing visualization of various parameters, such as pitch, loudness and formants (Timmers & Sadakata, 2014). In this way, additional information is included in the feedback learning process through visual presentation. The specific visualization can focus the user’s attention towards a specific aspect of the sound, informing the user of specific aspects of performance that could be improved and potentially offering a method to increase perceptual sensitivity. Learning efficacy depends crucially on the type of feedback, the complexity of the task, and the individuals’ skill level (Wilson, Lee, Callaghan & Thorpe, 2008; Rossiter & Howard, 1996; Brandmeyer, Timmers, Sadakata, & Desain, 2011). This interaction seems to hold more generally for motor skill acquisition (Schmidt & Lee, 2010) and this makes it challenging to define a general rule to optimize feedback features. Therefore, fine-tuning of feedback features for the target task and population is necessary to maximize learning.

**Fig. 1:**
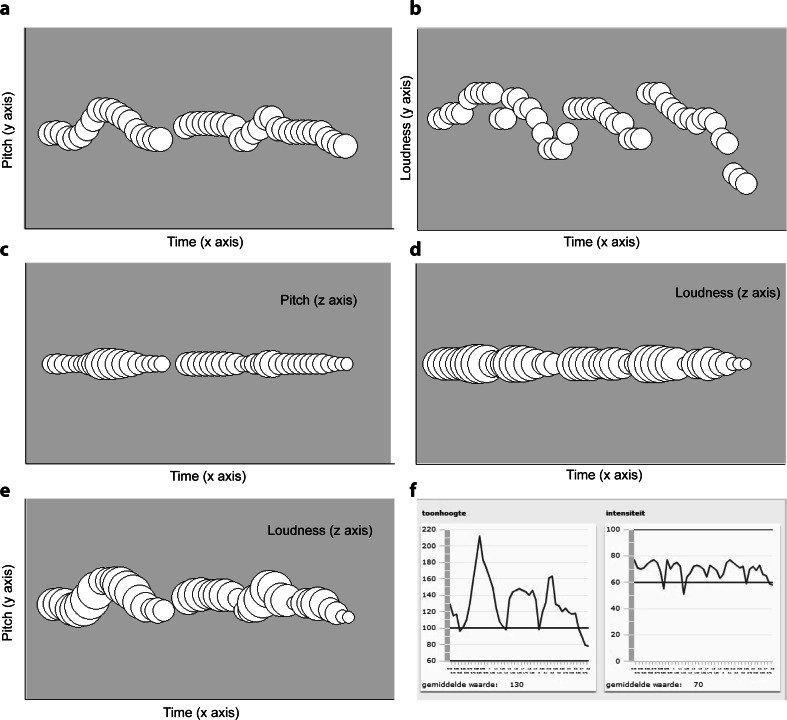
Stimulus visualizations. Time is always represented on the *x*-axis, and pitch and loudness are represented either on the *y*- or *z*-axes (**a**–**d**) or both (**e**). The original static feedback used in the e-learning based speech therapy (EST) system is also shown (**f**)

Investigations into e-learning based speech therapy (EST, Beijer et al., 2010b; Beijer, Rietveld, Hoskam, Geurts, & de Swart, 2010a) have shown that visualizing pitch height and loudness of speech helps patients in receiving meaningful computerized feedback based on personalized training goals, thus allowing efficient home-based practice. The visual feedback for this method was initially created as two separate graphs of pitch and loudness, plotted on the *y*-axis with time on the *x*-axis. Pitch and loudness are the main aspects of speech that need to be practiced in patients with Parkinson’s disease (PD), and the technique of pitch limiting voice treatment (PLVT), which aims to increase loudness while at the same time limiting an increase in vocal pitch to prevent a strained or pressed voice (de Swart, Willemse, Maassen & Horstink, 2003) is often employed. Findings from a case study (Beijer et al., 2010a) with a patient with PD indicated that visualization needed to be improved in terms of its interpretability (see Fig. 1f). The use of intuitive, integrated and informative visualization of speech is highly relevant to the effectiveness of the intervention; to assist patients in an independent web-based speech training process, the visualizations should be easy to understand, and make apparent sense for a particular sound. To this end, optimally intuitive mappings between visual and sound dimensions need to be established.

In the current work, several visualizations of speech were evaluated in terms of fit between auditory and visual dimensions. In the literature on crossmodal correspondence, a number of consistent mappings have been reported. The term crossmodal correspondence refers to congruency between dimensions in different sensory modalities, ranging from low-level amodal properties to more abstract, high-level cognitive correspondences based on stimulus meaning (Spence, 2011). With the majority of research focusing on the mappings between visual and auditory aspects, the use of static figures and relatively short sounds is most common, and has revealed associations of pitch height with vertical position, brightness or lightness of the stimulus and more angular, or smaller shapes, and of loudness with brightness (for more detail, see review by Spence, 2011). However, these studies have generally used categorical instances of visual or auditory dimensions (i.e. high and low pitch matched with small or large shapes) rather than continuous measures and sound sequences. This is not the case for studies looking at correspondences between visual features and musical fragments, which are more like speech in the sense that a longer, continuous sound is represented visually. Previous literature on visualizing musical aspects has also shown interesting cross-modal associations. For example, the so-called spatial-musical association of response codes (SMARC) effect describes a tendency that (musically trained) listeners associate high pitch tones with the right-up corner and low pitch tones with the left-bottom corner of a two-dimensional (2D) plane (Rusconi, Kwan, Giordano, Umiltá, & Butterworth, 2006). In line with this finding, among various visual features height is one of the most prominent dimensions to be associated with musical pitch (Eitan & Timmers, 2010; Küssner & Leech-Wilkinson, 2014; Lipscomb & Kim, 2004; Walker, Bremner, Mason, Spring, Mattock, Slater, & Johnson, 2009). Similarly, loudness of musical sound has been associated with the size of visual objects (Küssner & Leech-Wilkinson, 2014; Lipscomb & Kim, 2004; Nijs & Leman, 2014). These associations make intuitive sense, as we often refer to pitch as being high or low, and loudness and size could both indicate distance from an auditory object; however, to our knowledge they have not been evaluated for speech sounds.

In order to evaluate these findings for speech material, we devised multiple visualizations for spoken sentences, and asked participants to rate how well they thought the visualization fit with the sound (or if they preferred that visualization as the better match). Pitch was visualized either as the positioning of circles in the vertical dimension (*y*-axis), or as the size of a circle (or *z*-axis, taking size as the third dimension); the same was done for loudness. Time was always represented on the *x*-axis (left to right), thus creating an animation as the circles appear with the speech sounds. A stable mapping for time as left to right has been established for duration (Walker, 1987), early or late clicks [spatial temporal association of response code (STEARC) effect; Ishihara, Keller, Rossetti & Prinz, 2008], as well as for musical material (Athanasopoulos & Moran, 2013). In the interest of experiment time, the only combined visualization that was used represented pitch on the *y*-axis (vertical space) and loudness in circle size. Finally, the original visualization used in EST was also included, to provide a comparison with the current system (all visualizations are shown in Fig. 1).

The outcome measure, namely the perceived goodness of fit of the visualization with the sound, can be used to address a number of questions. Of these, the most relevant to speech therapy applications is which of the two important acoustic features for the PLVT method, namely pitch and loudness, fits better with the *y*- and *z*-axes (vertical position and size of visual objects). We hypothesized that the previously reported findings for music would be replicated for speech material, leading to pitch being associated most with the *y*-axis and loudness with the *z*-axis (cf. Eitan & Timmers, 2010; Küssner & Leech-Wilkinson, 2014; Lipscomb & Kim, 2004; Walker et al. 2009). A second question of interest is whether visualizing only one of the two features is enough to create a fitting impression, or if a combined visualization of the two would be evaluated as better. We predicted that visualizing pitch and loudness in combination would be the most fitting, as this is the most complete representation of the sound. Finally, by comparing the newly developed visualizations to the original EST visualization, we can compare the effect of presenting an animated visualization instead of a static image, as well as that of a single representation compared to two graphs.

## Method

### Participants

Participants were recruited online through global social and professional networks, mailing lists and web portals, and were given the option to select either English or Dutch as the instruction language. A total of 271 participants completed the web experiment on their own personal computer. Participants who reported neurological disorders or any uncorrected impairments in hearing or vision were excluded, leaving 249 participants (77 male and 172 female). Their age groups and choice of experiment instruction language are shown in Table 1. Almost one-half (41.8 %) of the participants who opted for English instructions indicated that English was not their mother tongue.

**Table 1:** Participants: age distribution and experiment language choice

Age group (years)	0-20	21-40	41-60	61-80
Dutch version	1	67	54	25
English version	1	70	23	8

### Stimuli

Six sentences were selected from a standard set of short Dutch sentences consisting of a noun phrase + verb phrase used in speech audiometry (Plomp & Mimpen, 1979), for example ‘In de *len*te lopen de *paar*den *heer*lijk in de wei (‘In spring, the horses run freely in the field’), with syllables in italics indicating pitch accents (associated with specific pitch movements) and potentially louder speech segments.

All stimulus sentences were spoken by a male speaker. The audio recordings had a sampling rate of 44.1 kHz and were high-pass filtered at 50 Hz. The visualizations were created by first estimating the pitch and loudness in the sound signal using the speech processing software ‘Praat’ (Boersma & Weenink, 2005), and removing all the zero values (which represent short silences). Then, outliers in pitch contours were removed using a 3rd-order median filter. The range of the pitch and loudness were normalized to fit the range of the visualization space (300 x 550 pixels) and the starting point on the *y*-axis was plotted at 10 % of the vertical space. The *z*-axis (or size) increases were implemented linearly.

Three experiment versions were created, containing two of the six selected sentences each, thus creating three different stimulus sets. Six visualizations were created for every sentence, varying the mapping to the vertical direction (*y*-axis) and size (*z*-axis) of circles appearing with the sound, shown in Fig. 1, with the horizontal direction fixed to represent time. Two visualizations used only the *y*-axis of the figure to visualize either pitch or loudness, referred to as PitchY and LoudnessY (Fig. 1a,b). Figure 1c and d show the same principle applied to the *z*-axis, referred to as PitchZ and LoudnessZ. Figure 1e shows a combined visualization in which pitch represented the *y*-axis and loudness the z-axis, referred to as YZ-Combined, and Fig. 1f shows the original static feedback used in the EST system. Example animations are available as supplementary material.

**Fig. 2:**
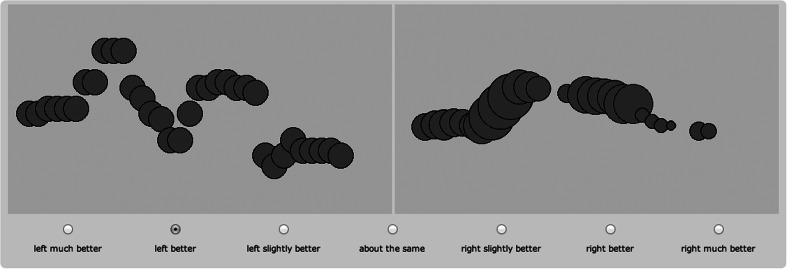
Example experiment screenshot showing LoudnessY and YZ-Combined visualizations, with clickable rating options below the two panels

All six visualizations were compared in all possible pairs and orders, yielding 15 combinations per sentence. Visualizing two sentences resulted in 30 comparisons per experiment version, taking 10–15 min in total. Figure 2 shows a screenshot of the web interface, where relative preference for the visualization displayed on either the left or right side of the screen could be indicated on a seven-point scale. The presentation of the comparisons was randomized. The order of presenting two visualizations (left/right) was also randomized, and the color of the shapes was varied randomly (but kept stable within comparisons).

### Procedure

Participants completed the web experiment on their own personal computer, after initially testing for sufficient sound volume. The use of headphones or earphones was strongly encouraged. After selecting Dutch or English as the preferred experiment language (note that all stimulus sentences were in Dutch), participants answered some basic questions regarding their demographic information and reporting of any perceptual or neurological problems. Participants were randomized over the three experiment versions based on their experiment language, gender and age category, creating optimally matched groups for each version.

Thirty pairs of visualizations (15 for each sentence) were shown in turn, allowing the participant to view two animations per comparison, with the final shapes remaining on screen while participants responded at their own pace. Participants were asked to rate which one of the two visualizations best matched the sound on a seven-point scale, indicating preference for either the left or the right option (see Fig. 2 for a screenshot of the experiment). Thus, a preference score is produced, on a scale ranging from −3 (stimulus A is greatly preferred to stimulus B) via 0 to +3 (stimulus B is greatly preferred to stimulus A), yielding distances between the compared visualizations.

After these 30 comparisons had been completed, another short list of questions was presented, asking participants about their experience during the experiment as well as additional questions about their mother tongue and understanding of the sentences. The experiment took about 15 min in total.

### Analysis

The scores of each comparison were analyzed using Scheffé’s paired comparisons test (Scheffé, 1952). This method was developed to procure a ranking of stimuli at an interval scale. Based on the variances and degrees of freedom in the data, a ‘yardstick’ is produced to determine a minimum distance in the ranking that denotes a statistically significant difference between objects (in this case visualizations), based on a chosen significance level. This method has been used previously in the study of intonation, in the evaluation of synthetic speech and in the assessment of intelligibility of dysarthric speakers (Beijer, Rietveld, Ruiter & Geurts, 2014). This analysis was performed for the entire participant group as well as for subsets of the group, yielding rankings for subgroups varying in experiment language, age groups, stimulus-set and gender. Statistical significance was tested at α = 0.01.

## Results

**Fig. 3:**
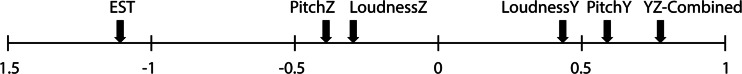
The full dataset ranking and distances according to Scheffe’s test of paired comparisons, with higher values representing increased preference. Preferred visualizations are ranked from high to low-matching as YZ-Combined, PitchY, LoudnessY, LoudnessZ, PitchZ, static graphs (EST)

The ranking and distances of the whole group are shown in Fig. 3. The preference ranking showed that the YZ-Combined visualization was by far the most preferred, and the static graphs used in the original version of EST the least preferred. The two visualizations using the *y*-axis were next highest in the ranking (first pitch, then loudness), followed by the *z*-axis (first loudness, then pitch). Table 2 summarizes the preference scores for all comparisons. For this dataset, the yardstick *Y*._01_ indicating a significant difference between the ratings of compared pairs was calculated to be 0.0171. All difference values exceeded 0.0171, indicating that the ratings of all comparisons differed significantly (*P* < .01). For various participant subgroups, separated for each experiment version (stimulus sentence set) or by demographic characteristics (gender, age and language), the distances varied slightly but the preference orders were all the same, with significant differences between all pairs, and are not further reported here.

**Table 2:** Summary of estimated differences between preference scores for all visualization comparisons (abbreviations described under ‘Stimuli’)

	PItchZ	LoudnessY	LoudnessZ	YZ-Combined	EST
PitchY	0.98	0.155	0.885	0.185	1.697
PitchZ	–	0.825	0.095	1.165	0.718
LoudnessY	–	–	0.730	0.340	1.543
LoudnessZ	–	–	–	1.071	0.812
YZ-Combined	–	–	–	–	1.883

## Discussion

The crossmodal mappings of auditory and visual parameters were evaluated for speech sounds, providing a first large-scale investigation of the relation between speech sounds and visual space, with implications for therapeutic paradigms meant to support speech therapy with visual feedback. Our results matched earlier findings of associations of visual dimensions with continuous (musical) sounds, on a large scale and with a wider age range. In terms of the visual dimensions that were used (*y*- and *z*-axes), the *y*-axis is rated to better represent both pitch and loudness aspects, with pitch rated as more fitting than loudness. However, on the (lesser-preferred) *z*-axis, loudness was judged as better fitting than pitch, which is also in line with the correspondence reported for static shapes where larger size is mapped to lower rather than higher pitch (cf. Gallace & Spence, 2006). The combined visualization was considered the best, and the static two-graph visualization was considered the worst fit. Although these findings were attained through a web-based experiment with less rigid experimental control, the relatively large test group (*n* = 249) and the replication of the preference rankings for different genders, age groups and stimulus materials supports the robustness of this result. Furthermore, the lack of discrepancy between the rankings obtained by English and Dutch version of experiments indicates that prosodic information could be judged independently from semantic information of the sentences used, which was not available to non-Dutch speaking participants. This supports the generally reported notion that some crossmodal correspondences, especially the more low-level perceptual mappings, are found across different cultures and are considered to be universal (cf. Walker, 1987; Spence, 2011, but see also Athanasopoulos & Moran, 2013, and Küssner, Tidhar, Prior & Leech-Wilkinson (2014) for influences of culture and training on reliability of crossmodal mappings).

These results essentially support but also refine our hypotheses, namely that in representing single dimensions (pitch height or loudness), the *y*-axis or vertical space is most associated with pitch, whereas size (or the *z*-axis) indeed fits best with loudness. However, for loudness the reverse does not hold, in that the general preference for the *y*-axis overrides the association of loudness with the *z*-axis, and thus LoudnessY is preferred over LoudnessZ, which was not predicted. This may be related to vertical space being used more commonly than size when visualizing a time course or changing signal, even though the classic visualization of sound waveforms (although not a veridical representation of perceptual loudness) visualize loudness much more obviously than pitch. The finding that the combined visualization was most preferred also supports our hypotheses, although in the absence of the reversed combined visualization (with loudness on the *y*-axis and pitch on the *z*-axis), this preference cannot be interpreted as a support for a specific mapping. In terms of the low preference for the static images, it must be noted that, although the aim was to provide a baseline score for the comparison between visualizations, it is likely that the simple fact that the other visualizations were animated precluded equal comparisons with the separate static graphs. However, in the interest of evaluating the EST speech therapy system as it has been developed, we opted to keep the original visualizations as the comparison. However, it is clear that single animated visualizations are much preferred over separate static images. This preference indicates that visualization of an evolving time structure, as is inherent to longer sound fragments, increases the congruence between the visual and auditory stimuli.

Crossmodal congruencies have been described as having several possible origins, ranging from structural correspondences, thought to be based on commonalities in neural processing, to statistical correspondences, based on consistent co-occurrence of stimulus attributes in the environment, to semantically mediated correspondences, based on common linguistic terms (see Spence, 2011, for further discussion). In the context of the current findings, there is no possibility of distinguishing adequately between these phenomena, as arguments can be made for multiple mechanisms. Behavioral findings have suggested that the cross-modal mapping between pitch and vertical orientation is innate (Walker et al., 2009). However, a low-level perceptual correspondence may well be further strengthened by the use of the words ‘high’ and ‘low’ for pitch; both have semantic and spatial implications, see for example Dolscheid, Shayan, Majid & Casasanto, (2013) for an elegant demonstration of how changing linguistic space-pitch metaphors can impact representations of pitch. The correspondence of large objects and loud sounds could be explained by the common occurrence of previous experience of this mapping, as well as by the inference that physically bigger (or closer) beings or objects often make louder sounds than smaller (or more distant) beings or objects. The preference for the *y*-axis over the *z*-axis for feature representation may also indicate that information is transmitted more easily in this dimension, which in this case might be related to the scale used in each dimension; in the current setting, the *y*-axis necessarily had a greater range of display.

There are some limitations of these results in terms of generalizing our findings to practice, for example speech therapy for PD patients as reported in the original EST study by Beijer et al. (2010a), or other paradigms that make use of visual feedback of sound. Additional research will need to show that the current findings also hold for the targeted user groups, who may have specific attentional or perceptual deficits. For instance, previous research investigating pure tone discrimination in PD patients showed a reduced ability to notice change in frequency (or pitch) and intensity (or loudness) as compared to healthy older adults (Troche, Troche, Berkowitz, Grossman & Reilly, 2012). Results of a study into auditory speech discrimination by means of paired comparisons also showed problems in detecting different frequency levels, but not in different intensity levels (Beijer, Rietveld & van Stiphout, 2011). PD patients may also be impaired in judging loudness in their own speech (Ho, Bradshaw and Iansek, 2000). These perceptual deficits are relevant to the design of possible visualizations for speech therapy for PD, as visual feedback can be used to highlight acoustic features that provide important learning information, but are not easily perceived. Of course, other user groups or specific applications of the feedback learning paradigm may necessitate a different set of visualization criteria and paradigm features. Additionally, it should be noted that preferred visualizations are not necessarily more useful while extracting information about the sound. For example, Brandmeyer et al. (2011) found that, although the majority of participants preferred an analytic, informative visualization of music performance, the most useful visualization in terms of learning performance was holistic, without explicit information. Now that we have established the most intuitive way is to represent pitch and loudness of speech in an animation, the next step is to validate the transfer of information about aspects of speech that need to be changed, namely, pitch and loudness. In this way, visualizations can be developed that are not only intuitive but also maximally informative.

The current study contributes a large-scale investigation of the preferred mappings of speech sounds to visual dimensions, which turn out to be generalizable over age groups and gender, and independent of semantic understanding or specific sentences. The results extend previous reports for the musical domain (Lipscomb & Kim, 2004; Küssner & Leech-Wilkinson, 2014), and complements work on more general associations of auditory and spatial mappings in movement (Eitan & Granot, 2006; Burger, Thompson, Luck, Saarikallio & Toiviainen, 2013). However, as Eitan & Timmers (2010) note, it is possible that other visual and metaphorical features interact with how one represents cross-modal mapping and this should be further refined.

Although additional work will be necessary to ascertain the ideal parameters for the various clinical and non-clinical populations for whom this method may be relevant, animated, combined visualizations are robustly found to be most fitting for speech sounds in healthy participants. Clearly, the application is not limited to speech therapy for PD, but may be extended to other rehabilitation or learning goals. Within speech therapy contexts, this method may be extended to groups experiencing dysarthric speech resulting from varying underlying neurological problems (cf. Kim et al., 2011), but the method may also benefit individuals with hearing disorders. Non-clinical feedback-based learning paradigms that involve speech, such as learning formants or tonal aspects in second language learning and music pedagogy applications that make use of corresponding mappings (e.g., de Bot, 1983; Hoppe, Sadakata & Desain, 2006; Nijs & Leman, 2014), could also benefit from these findings. Furthermore, the investigations of other possible mappings of sound aspects (spectral content, aspiration, attack and decay times and so on) to visual aspects (shape, color, brightness and more) offer many more possibilities to fully utilize the auditory and visual domains for feedback learning-based applications.

## References

[CR1] Athanasopoulos G, Moran N (2013). Cross-cultural representations of musical shapes. Empirical Musicology Review.

[CR2] Beijer LJ, Rietveld ACM, Hoskam V, Geurts ACH, de Swart BJM (2010). Evaluating the feasibility and the potential efficacy of e-learning-based speech therapy (EST) as a web application for speech training in dysarthric patients with Parkinson’s disease: a case study. Telemedicine Journal and E-Health.

[CR3] Beijer LJ, Rietveld ACM, van Beers MMA, Slangen RML, van den Heuvel H, de Swart BJM, Geurts ACH (2010). E-learning-based speech therapy: a web application for speech training. Telemedicine Journal and E-Health.

[CR4] Beijer LJ, Rietveld ACM, Ruiter MB, Geurts ACH (2014). Preparing an E-learning-based Speech Therapy (EST) efficacy study: identifying suitable outcome measures to detect within subject changes of speech intelligibility in dysarthric speakers. Clinical Linguistics & Phonetics.

[CR5] Beijer LJ, Rietveld ACM, van Stiphout AJL (2011). Auditory discrimination as a condition for E-learning based Speech Therapy: a proposal for an Auditory Discrimination Test (ADT) for adult dysarthric speakers. Journal of Communication Disorders.

[CR6] Boersma, P., & Weenink, D. (2005). Praat: doing phonetics by computer (Version 4.3.01) [Computer program]. Accessed online: http://www.praat.org/

[CR7] Bot de, K. (1983). Visual feedback of intonation I: Effectiveness and induced practice behavior. *Language and Speech, 26,* 331–350. 10.1177/002383098302600402 6677828

[CR8] Brandmeyer A, Timmers R, Sadakata M, Desain P (2011). Learning expressive percussion performance under different visual feedback conditions. Psychological Research.

[CR9] Burger B, Thompson MR, Luck G, Saarikallio S, Toiviainen P (2013). Influences of rhythm- and timbre-related musical features on characteristics of music-induced movement. Frontiers in Psychology.

[CR10] Demenko G, Wagner A, Cylwik N (2010). The use of speech technology in foreign language pronunciations training. Archives of Acoustics.

[CR11] Dixon S (2007). Evaluation of the audio beat tracking system BeatRoot. Journal of New Music Research.

[CR12] Dolscheid S, Shayan S, Majid A, Casasanto D (2013). The thickness of musical pitch: psychophysical evidence for linguistic relativity. Psychological Science.

[CR13] Eitan Z, Granot RY (2006). How music moves: musical parameters and listeners’ images of motion. Music Perception.

[CR14] Eitan Z, Timmers R (2010). Beethoven’s last piano sonata and those who follow crocodiles: cross-domain mappings of auditory pitch in a musical context. Cognition.

[CR15] Gallace A, Spence C (2006). Multisensory synesthetic interactions in the speeded classification of visual size. Perception & Psychophysics.

[CR16] Ho AK, Bradshaw JL, Iansek R (2000). Volume perception in Parkinsonian speech. Movement Disorders.

[CR17] Hoppe D, Sadakata M, Desain P (2006). Development of real-time visual feedback assistance in singing training: a review. Journal of Computer Assisted Learning.

[CR18] Ishihara M, Keller PE, Rossetti Y, Prinz W (2008). Horizontal spatial representations of time: evidence for the STEARC effect. Cortex.

[CR19] Kim Y, Kent RD, Weismer G (2011). An acoustic study of the relationships among neurologic disease, dysarthria type and severity of dysarthria. Journal of Speech, Language and Hearing Research.

[CR20] Küssner MB, Leech-Wilkinson D (2014). Investigating the influence of musical training on cross-modal correspondences and sensorimotor skills in a real-time drawing paradigm. Psychology of Music.

[CR21] Küssner, M. B., Tidhar, D., Prior, H. M. & Leech-Wilkinson, D. (2014). Musicians are more consistent: gestural cross-modal mappings of pitch, loudness and tempo in real-time. *Frontiers in Psychology, 5*, Art. 789. doi: 10.3389/fpsyg.2014.00789 10.3389/fpsyg.2014.00789 PMC4112934 25120506

[CR22] Lipscomb, S. D., & Kim, E. M. (2004). Perceived match between visual parameters and auditory correlates: an experimental multimedia investigation. In: Lipscomb, S., Ashley, R., Gjerdingen, R. & Webster, P. (Eds.), *Proceedings of the 8th International Conference on Music Perception and Cognition (ICMPC8),* pp. 72–75. Evanston, IL, 3–8 August, 2004. Adelaide: Causal Productions.

[CR23] McLeod, P. (2008). Fast, accurate pitch detection tools for music analysis. Unpublished PhD thesis. Department of Computer Science, University of Otago, 2008. Accessed 7 March 2012 at http://miracle.otago.ac.nz/tartini/papers.html

[CR24] Nijs L, Leman M (2014). Interactive technologies in the instrumental music classroom: a longitudinal study with the Music Paint Machine. Computers & Education.

[CR25] Plomp R, Mimpen AM (1979). Improving the reliability of testing the speech reception threshold for sentences. Audiology.

[CR26] Rossiter D, Howard DM, DeCosta M (1996). Voice development under training with and without the influence of real-time visually presented biofeedback. Journal of Acoustical Society of America.

[CR27] Rusconi E, Kwan B, Giordano BL, Umiltá C, Butterworth B (2006). Spatial representation of pitch height: the SMARC effect. Cognition.

[CR28] Sadakata M, Hoppe D, Brandmeyer A, Timmers R, Desain P (2008). Real-time visual feedback for learning to perform short rhythms with expressive variations in timing and loudness. Journal of New Music Research.

[CR29] Scheffé H (1952). An analysis of variance for paired comparisons. Journal of the American Statistical Association.

[CR30] Schmidt RA, Lee TD (2010). Motor control and learning: a behavioral emphasis.

[CR31] Spence C (2011). Crossmodal correspondences: a tutorial review. Attention Perception Psychophysics.

[CR32] Stowell D., & Plumbley, M.D. (2007). Adaptive whitening for improved real-time audio onset detection. In: *Proceedings of the International Computer Music Conference (ICMC’07)*. Vol 18. Denmark, August 2007.

[CR33] Swart de, B. J., Willemse, S. C., Maassen, B. A., & Horstink, M. W. (2003). Improvement of voicing in patients with Parkinson’s disease by speech therapy. *Neurology, 60*(3), 498–500. doi:10.1212/01.WNL.0000044480.95458.56 10.1212/01.wnl.0000044480.95458.56 12578936

[CR34] Timmers R, Sadakata M, Fabian D, Timmers R, Schubert E (2014). Training expressive performance by means of visual feedback: existing and potential applications of performance measurement techniques. Expression in Music Performance.

[CR35] Troche J, Troche MS, Berkowitz R, Grossman M, Reilly J (2012). Tone discrimination as a window into acoustic perceptual deficits in Parkinson’s disease. American Journal of Speech-Language Pathology.

[CR36] Walker P, Bremner JG, Mason U, Spring J, Mattock K, Slater A, Johnson SP (2009). Preverbal infants’ sensitivity to synaesthetic cross-modality correspondences. Psychological Science.

[CR37] Walker R (1987). The effects of culture, environment, age and musical training on choices of visual metaphors for sound. Perception & Psychophysics.

[CR38] Watanabe A, Tomishige S, Nakatake M (2000). Speech visualization by integrating features for the hearing impaired. IEEE Transactions on Speech and Audio Processing.

[CR39] Wilson PH, Lee K, Callaghan J, Thorpe CW (2008). Learning to sing in tune: does real-time visual feedback help?. Journal of Interdisciplinary Music Studies.

